# Knowns and Unknowns of Assaying Antibody-Dependent Cell-Mediated Cytotoxicity Against HIV-1

**DOI:** 10.3389/fimmu.2019.01025

**Published:** 2019-05-10

**Authors:** George K. Lewis, Margaret E. Ackerman, Gabriella Scarlatti, Christiane Moog, Marjorie Robert-Guroff, Stephen J. Kent, Julie Overbaugh, R. Keith Reeves, Guido Ferrari, Bargavi Thyagarajan

**Affiliations:** ^1^Division of Vaccine Research, Institute of Human Virology, University of Maryland School of Medicine, Baltimore, MD, United States; ^2^Thayer School of Engineering, Dartmouth College, Hanover, NH, United States; ^3^Viral Evolution and Transmission Unit, Department of Immunology, Transplantation and Infectious Diseases, IRCCS San Raffaele Scientific Institute, Milan, Italy; ^4^INSERM U1109, Fédération Hospitalo-Universitaire (FHU) OMICARE, Fédération de Médecine Translationnelle de Strasbourg (FMTS), Université de Strasbourg, Strasbourg, France; ^5^Vaccine Branch, Center for Cancer Research, National Cancer Institute, National Institues of Health, Bethesda, MD, United States; ^6^Department of Microbiology and Immunology, The University of Melbourne, at the Peter Doherty Institute for Infection and Immunity, Melbourne, VIC, Australia; ^7^Division of Human Biology, Fred Hutchinson Cancer Research Center, Seattle, WA, United States; ^8^Center for Virology and Vaccine Research, Beth Israel Deaconess Medical Center/Harvard Medical School, Boston, MA, United States; ^9^Department of Surgery and Duke Human Vaccine Institute, Duke University Medical Center, Durham, NC, United States; ^10^Global HIV Vaccine Enterprise, New York, NY, United States

**Keywords:** HIV—human immunodeficiency virus, antibodies, effector function, ADCC—antibody dependent cellular cytotoxicity, Fc receptor

## Abstract

It is now well-accepted that Fc-mediated effector functions, including antibody-dependent cellular cytotoxicity (ADCC), can contribute to vaccine-elicited protection as well as post-infection control of HIV viremia. This picture was derived using a wide array of ADCC assays, no two of which are strictly comparable, and none of which is qualified at the clinical laboratory level. An earlier comparative study of assay protocols showed that while data from different ADCC assay formats were often correlated, they remained distinct in terms of target cells and the epitopes and antigen(s) available for recognition by antibodies, the effector cells, and the readout of cytotoxicity. This initial study warrants expanded analyses of the relationships among all current assay formats to determine where they detect overlapping activities and where they do not. Here we summarize knowns and unknowns of assaying ADCC against HIV-1.

## Introduction

That Fc-mediated effector function contributes to antibody-mediated protection against HIV-1 for both broadly neutralizing antibodies (bnAbs) as well as non-neutralizing antibodies has become well-accepted. Although there are several categories of Fc receptors, this report is focused on the Fc-gamma receptors (FcγR) that are expressed largely on cells of the hematopoietic lineage including, B-cells, T-cells, monocytes/macrophages, dendritic cells, NK cells, and granulocytes, as well as on follicular dendritic cells of mesenchymal origin. FcγR play a pivotal role in coupling adaptive antibody (Ab) responses with innate immune effector responses by the recognition of antigen-antibody complexes (i.e., immune complexes, IC). Effector cell recognition by FcγR of IC formed on the surfaces of viruses, bacteria, and eukaryotic cells can result in their elimination by various mechanisms. In addition, FcγR recognition of IC on follicular dendritic cells as well as on B-cells plays a key role in the regulation of Ab responses. Thus, FcγR-mediated effector functions very likely play a pivotal role in vaccine-elicited protection against HIV-1 as well as in the prophylaxis and treatment of HIV-1 infections with Abs. Despite their apparent importance, there is still no consensus about which types of FcγR-mediated effector functions contribute to vaccine-elicited protection against HIV-1.

ADCC is characterized by IC coupled interactions between an effector cell and target cell that leads to target cell death. IC coupling occurs *via* the interaction of the Ab Fc region and the FcR on the effector cell and the Ab Fab region with antigen on the target cell. This interaction typically triggers the release of cytotoxic granules containing perforin and granzymes from the effector cells to the target cell *via* an immunologic synapse resulting in target cell lysis. ADCC has been correlated with vaccine-elicited protection in non-human primates (NHPs), reduced risk of infection/mortality in the setting of mother to child transmission, and in the RV144 human vaccine trial, where it emerged as a secondary correlate of reduced infection risk [(1–5), reviewed in (6)]. These observations place ADCC at the forefront as a potential correlate and mechanism of protection against HIV-1. Although the data strongly suggest a role in Ab-mediated protection against HIV-1, questions remain about which of the many ADCC assay formats best reflects the biology of protection and therefore should be used in clinical trials of HIV-1 vaccines. This issue is complicated by the necessity of the assay to be amenable to qualification as a clinical assay in a high-throughput format.

## Complexities of Measuring ADCC

**Figure 1:**
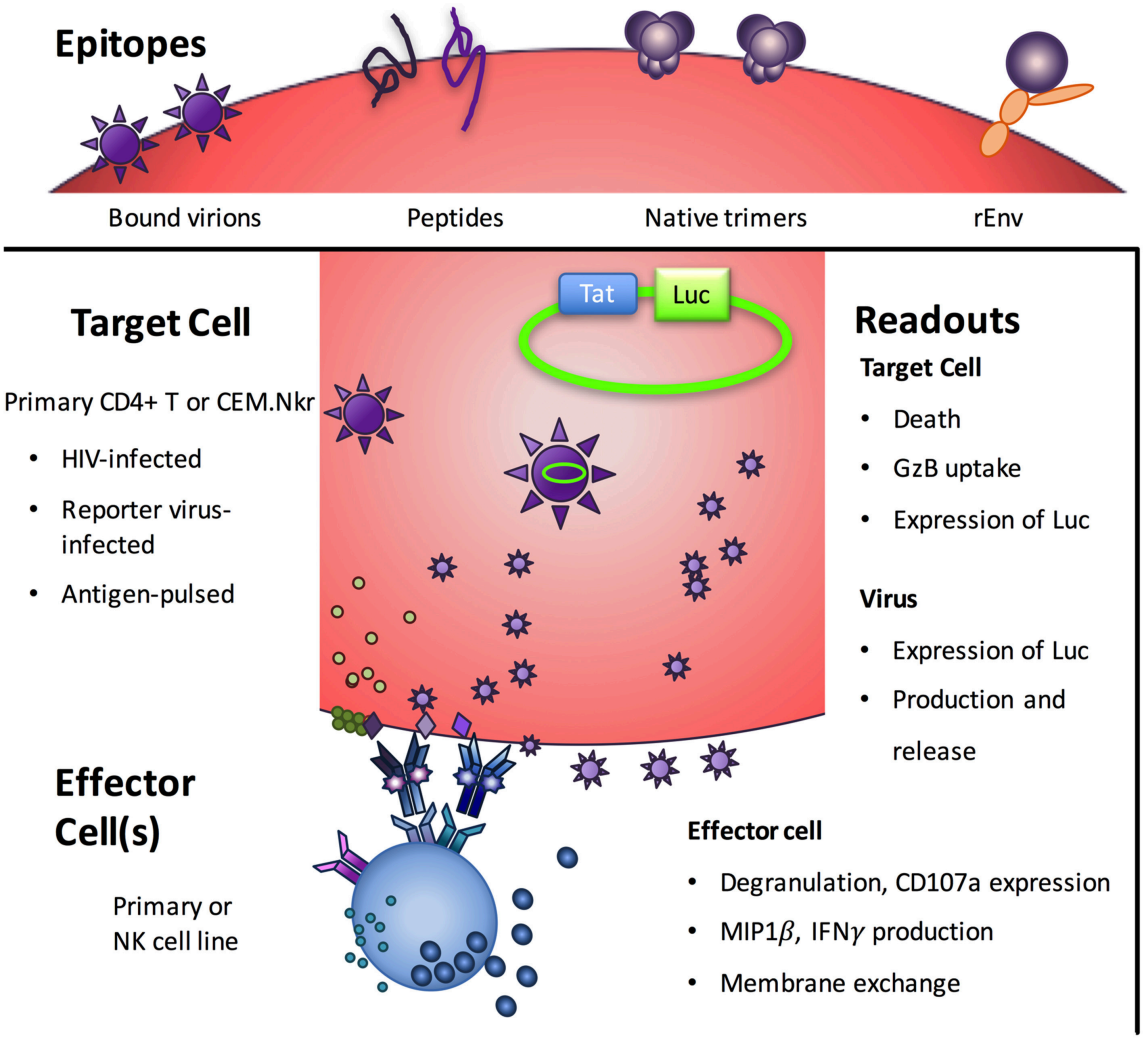
Notable variables among ADCC assays. Beyond the monoclonal or polyclonal antibodies being assessed, ADCC assays vary in terms of the viral epitopes presented, the target cells on which they are presented, the effector cells that will respond, and the readout of the biological activity assayed. Image adapted from (7).

At the minimum, an ADCC assay requires an effector cell, an antigen-bearing target cell, Abs, and a means to measure cytotoxicity. These nominal requirements veil the true complexity of ADCC assays and the biology they are thought to represent. For example, there are major differences among the various assay formats in effector cell type, target cell type, antigen targets, and the readout of activity (Figure 1 and Table 1).

**Table 1:** Cell-based ADCC assay variables with exemplary references.

**Effector cells**	**Target cells**	**Target antigen**	**Readout**
**Primary cells** PBMCs (8, 9) NK Cells (10) Monocytes/Macrophages (11) Neutrophils (12, 13)	CEM.NKR_CCR5_ Primary activated CD4+ T cells	**HIV-1 Infected Cells** Native Trimeric Env (14, 15) CD4-trigggered Env (Nef/Vpu modulated) (16, 17) **Other** Env Transfected Cells (31) gp120-coated cells (9, 32) Peptide-coated cells (20) Inactivated virus-treated cells (33)	^51^Cr Release (18) Dye Loss (8) Dye Uptake (19) Granzyme Transfer (20, 21) Reporter Gene Loss (15) IFN-γ Intracellular Staining (20) CD107a down regulation (22) Ligand Transfer (trogocytosis) (23) Intracellular p24 antigen staining (24, 25) Reduction in virus production (26)
**Cell lines** FcγR Transduced KHYG-1 NK Cell (15) THP-1 Monocytic Cell (27) FcγR+ Jurkat T Cell (28–30)			

A major point of differentiation among ADCC assay formats is how target cells present antigen, which has a major impact on the specificity of Ab responses detected by the assay. Some assays use target cells incubated with recombinant extracellular domains of envelope protein trimers or gp120/140 subunits, or even peptides (8, 20). While there may be more than one means of association between envelope proteins in such “coated” cells, the interaction between recombinant envelope and cells is thought to primarily be achieved *via* direct interaction with CD4, resulting in CD4-induced conformational changes. In contrast, assays utilizing inactivated virus, reporter viruses, and infectious virions differ in a number of additional ways with respect to their antigenic composition. Infection of target cells with unmodified infectious virus will downregulate CD4 from the cell surface, supporting presentation of “native trimer” envelope conformations, as opposed to CD4-induced (CD4i) monomeric conformational states. Relative to “coated cell” assays, those that employ reporter viruses can have the advantage of presenting full envelope glycoprotein with native transmembrane domains and associated epitopes. However, these epitopes too may differ from unmodified viruses in their level of envelope expression and their ability to drive CD4-downregulation, resulting again in presentation of different conformational states of envelope, and sensitivity to different antibodies. Further, CD4 receptor downregulation, envelope expression, virus budding, and envelope shedding are all time-dependent processes, complicating comparison of readouts from different ADCC assay protocols. It is possible that the spectrum of envelope states on infecting and/or budding virus relevant to anti-viral activity *in vivo* may be quite broad. As one specific example, longitudinal exposure of infected cells to Dual-Affinity Re-Targeting (DART) molecules derived from combinations of anti-HIV-1 non-neutralizing and anti-CD3 targeting antibody Fabs resulted in CD3+ T cell mediated killing even when surface expression of Env appeared low (34). Thus, it is clear that there is a rich milieu of different viral epitopes addressed across different assay types and over different time scales.

In addition to a multitude of Env-bearing target cells studied, ADCC assays commonly employ different effector cells. These range from NK cell lines to mixed populations of primary peripheral blood mononuclear cells (PBMCs). This variety of effector populations expresses different levels and compositions of antibody receptors including FcγR and FcαR, as well as accessory proteins involved in downstream signaling and biological activities. Even among NK cells, expression of higher and lower affinity polymorphic variants of FcγRIIIa, at different levels and in the context of different signaling partners (35, 36), are known to impact outcomes in ADCC assays. PBMC-based assays will include monocyte populations expressing FcγRIIa and FcγRIIb receptors that (a) have different preferences among IgG subclasses and Fc glycoforms; (b) are considered activating and inhibitory, respectively (37–39), and; (c) vary in allotypic composition of FcγR from donor to donor. Beyond inherent differences in receptor expression and activities, effector cells are present at different sites at different levels, and tissue localization can change FcγR expression levels, activation status, and functional competence (40–42). Assays are often conducted using mixed PBMCs, purified primary cell subsets, and/or cell lines; yet, these cells may not accurately reflect the activities most relevant to tissue-resident effector function *in vivo*, across different sites and local environmental cues. Activity can also be affected by other, less obvious factors. For example, expression of FcαR and the presence of IgA that binds to but does not activate FcαR has been reported to interfere with responses from activating FcγR (43–45). In contrast, IgA and its receptors can also drive effector function (46–48). The complexity of this biology is perhaps most simply demonstrated by the observation that both the inhibitory and activating roles of FcαR rely on the common γ chain, which is also critical to FcγR-mediated activities. However, the potential relevance of such nuanced cell biology to outcomes of HIV vaccination can be found in observations that IgA can interfere with IgG-mediated ADCC (49, 50), and that IgA and ADCC were observed to have opposing relationships to risk of infection among vaccine recipients in the RV144 vaccine trial (3) whereas IgA has shown positive associations with protection in NHP vaccine models (46, 51).

In addition, assayed outcomes differ significantly across approaches. They include readouts associated with target cells and readouts of effector cells. Among target cell endpoints, Cr^51^ release, dye release, and reporter loss have all been assayed. Among effector cells, dye uptake, CD107a staining, MIP1α and IFNγ production, granzyme transfer, and ligand transfer, among others have been assayed. Further, even outcomes such as reduction of virus outgrowth have been utilized (26). Overall, given this variation in (a) presentation of antigen epitopes and conformations on target cells, (b) polymorphisms, levels, and composition of expressed FcγR and downstream signaling partners, within and among distinct effector cell types with different preferences among antibody subclasses and glycoforms, and (c) endpoints alternatively focused on target or effector cells relating activities ranging from target cell death to expression of cytokines from effector cells, rich insights into these aspects of immunobiology result from analysis of various monoclonal and polyclonal antibody samples across studies.

## Impact of Vaccine-Induced ADCC Activity on Protective Efficacy in Non-Human Primates; Early Studies

**Figure 2:**
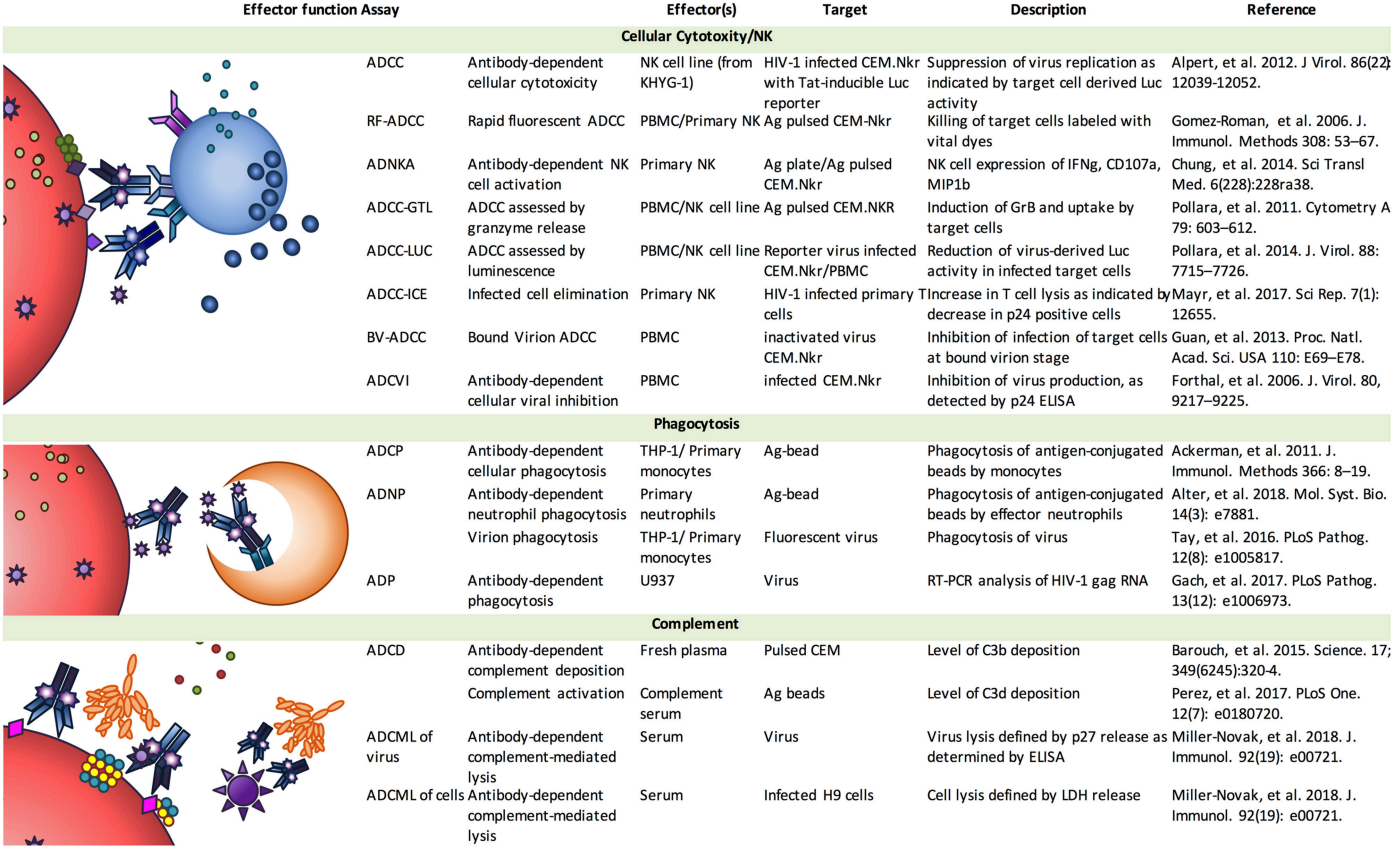
Effector function assays. Antibodies elicit the activities of a diverse array of effector cell types and mechanisms, leading to the generation of a variety of *in vitro* assays aimed at characterizing the activity of mAb and pAb samples, and defining further insights into basic mechanisms. Image adapted from (7).

Early work reported consistent correlations between ADCC and vaccine-elicited protection against SIV and SHIV in NHP models. This work was principally accomplished using the rapid fluorometric antibody-dependent cellular cytotoxicity assay (RFADCC) (Figure 2) (8), in which CEM.Nkr (NK-resistant) target cells are double-stained with a membrane and a viability dye, and following incubation with antibodies and PBMCs as effector cells, target cell death is assessed by flow cytometry and quantified as the fraction of non-viable target cells. This assay continues to be used widely to evaluate ADCC. Correlations between ADCC titers and protection were observed in both single high-dose and repeat low-dose SIV challenge studies. In the single high-dose SIV challenge studies, the NHP were not protected from infection, but post-infection control of viremia, correlated with ADCC, was observed in the vaccine groups across several studies (52, 53). Using repeat low-dose challenge protocols, which are thought to more accurately reflect sexual transmission in humans, vaccinated NHP resisted infection with SIV. This protection against infection also correlated with ADCC (54). Taken together, the body of literature developed by Dr. Robert-Guroff and her colleagues strongly suggests that ADCC assessed by the RFADCC method correlates with vaccine-elicited protection in NHP models of infection and supports further exploration of ADCC in large-scale HIV-1 vaccine trials.

## Inverse Correlations Between ADCC and Breast Milk Transmission of HIV-1

Recent studies from the Nairobi breastfeeding clinical trial showed that passively transferred ADCC-mediating Abs correlated with favorable infant outcomes (5). In the absence of treatment, approximately 40% of infants exposed to HIV-1 become infected suggesting that there may be factors that protect some infants from infection. Using the RFADCC assay and target cells coated with gp120, Milligan et al. (5) showed a trend for higher passively transferred ADCC activity in infants who don't acquire HIV-1. In the subset of infants who acquired HIV-1 infection, there was a significant correlation between passively acquired ADCC-mediating Ab and the survival of infected infants. By contrast, there was no correlation between infant infection or survival outcomes and passively acquired neutralizing Abs in the same cohort (5, 55). The levels of HIV-specific IgG in the infants were also not correlated with these outcomes, suggesting the effect was specific to ADCC-mediating Abs.

Another study from the Nairobi trial showed that among mothers who would be expected to be highly infectious based on having high viral loads, non-transmitting mothers had higher ADCC titers in their breast milk as compared with transmitting mothers, lending further support that ADCC activity as measured by RFADCC predicts outcome (4). These studies are among the first to show an immune correlate of protection from HIV infection in human studies and suggest the need for a more detailed evaluation of the specificity and function of Abs that could contribute to protection in the setting of mother-to-child transmission.

## Functional Cytotoxicity-Based Assays to Detect Vaccine-Induced ADCC Responses in Clinical Trial Settings

The measurement of ADCC activity in a clinical trial setting requires more rigorous standardization than is needed for a research laboratory. This problem was addressed in an ADCC comparative study (56). To date, the most experience using ADCC in a clinical trial setting resides in the NIAID sponsored HIV Vaccine Clinical Trials Network (HVTN) where extensive and specific quality control criteria have been developed and implemented to perform two ADCC assay formats that permit rigorous comparisons among independent human HIV-1 vaccine trials. One assay, denoted as the GranToxiLux (GTL) assay (57), measures the transfer of granzyme from the effector cell to the target cell as a surrogate of NK cell-mediated lysis. The platform utilizes gp120-coated target cells, which have been historically utilized as target cells to detect anti-HIV-1 ADCC responses (9). Moreover, the ADCC responses detected with gp120-coated target cells have been correlated with vaccine-induced Ab responses that can control virus replication (52, 53, 58, 59) and prevent infection (1, 2, 54, 60) in pre-clinical studies as well as with prevention from mother to infant transmission of HIV-1 (4). The assay may represent a surrogate of the CD4 T-cells targeted by ADCC-mediating Abs during virus entry at the time of gp120-CD4 receptor engagement as suggested by the correlation between results generated by this assay and an ADCC assay that utilizes virus-bound target cells (33, 56). Because the recombinant gp120 protein interacts with target cells *via* CD4, this assay cannot measure Ab responses recognizing the CD4 binding site (CD4bs), but it can detect those directed against CD4 inducible epitopes (CD4i). Moreover, whole PBMC were used as source of the effector population to generate data with GTL assay and area scaling analysis was applied (57) to directly quantify the contributions of NK cells vs. monocytes that recognize the target cells based on the frequency of Granzyme B+ events within singlet and doublet populations representing cells recognized by the NK cells and monocytes, respectively. Such de-convolution of effector cell types demonstrated the correlation between NK cell-mediated ADCC activity and protection in a NHP vaccination/challenge study (1, 57).

Another assay, denoted the Luciferase-based (Luc) ADCC assay (Figure 2), utilizes target cells that are infected with HIV-1 Infectious Molecular Clones (IMC), expressing a Luciferase reporter gene under the control of HIV-1 Tat, which allows for detection of target cell elimination following the infection of cells and virus replication (61). The final read-out is based on the reduction of luciferase signal upon incubation of target and effector cells in presence of a source of Ab. During virus replication, diverse conformations of the HIV-1 envelopes are presented on the membrane of the infected cells including exposure of CD4bs epitopes as well as those represented by closed Env trimers. Of note, for qualification purposes of this assay under Good Clinical Laboratory Procedures (GCLP) guidelines, it was observed that the median level of CD4 downregulation was 56% (range 39–83%) and 69% (range 34–89%) at 48 and 72 h post-infection. The levels of CD4 downregulation and frequency of CD4+ infected cells observed in these target cells were comparable to those observed in primary CD4+ T cells infected with primary HIV-1 isolates reported by different groups (62–64). The 48 and 72 h post-infection times were defined as optimal to allow for maximum virus replication before initiating the incubation of IMC-infected cells with the Ab sample of interest to detect ADCC responses. Under these experimental conditions, the lower level of Nef expression in the CEM.Nkr was compensated by the Vpu in the 2TA reporter IMCs to achieve downregulation of CD4 on the infected cells, as further discussed below. With this assay, it was shown that susceptibility to ADCC does not cluster based on Env subtype, instead, it appears that there is a tiered ranking of ADCC responses for CEM.Nkr infected with different IMCs of HIV-1 (65). Moreover, the tiered ADCC ranking was distinct from the tiered ranking widely used for neutralization of HIV-1 with the Tzm-bl assay, illustrating that these two assays detect significantly different biological responses.

## Deciphering ADCC Activity on Primary Infected Cells

One recurrent question is how different ADCC assays recapitulate *in vivo* lysis of infected cells. Most studies of ADCC have employed various target cell lines with the most frequently used being variants of the CEM.Nkr T-cell line and diverse effector cells such as primary NK cells, primary monocytes, PBMCs, and NK cell lines (Table 1). Some studies have articulated the confounding effects of uninfected bystander cells (66), and the effect of different viral backgrounds that may or may not be fully replication competent or express fully functional Nef and Vpu accessory proteins (16, 66–71). Of note, introduction of the Luciferase reporter into the IMC construct can affect down-regulation of CD4 by Nef (69), but does not impact the Vpu-mediated down-regulation (72). Therefore, the time- and replication-dependent down-regulation of CD4 must be carefully evaluated using these assays as they are also influenced by the type of target cells used, i.e., cell line vs. activated primary CD4 T-cells.

Therefore, while at odds with attributes needed for implementation in large scale evaluation of vaccine-elicited responses, having a fully autologous assay system comprised of primary HIV-1 infected cells and primary effector cells from the same donor may give a more physiologically relevant picture of the true function of Abs with ADCC activity. To this end, an assay using autologous PBMCs infected *in vitro* with HIV-1 as targets and NK cells purified from these PBMC as effectors has been developed (24). This ADCC system measured the increase in lysis observed in the presence vs. absence of NK cells, and was compared with the NK-mediated ADCC assay using HIV-1 infected CEM.Nkr cells and the NK cell CD107a expression ADCC assay using monoclonal Abs and polyclonal antisera from HIV-1 infected subjects (20). Strikingly, ADCC under these potentially physiologically more relevant conditions (i.e., the primary autologous system) was distinct from that obtained with the assay format using HIV-1 infected CEM.Nkr cells.

Interestingly, non-neutralizing monoclonal Abs directed against the V2 loop, that were previously found to be associated with vaccine-elicited decreased risk of infection in the RV144 vaccine trial, showed highly efficient ADCC activity under these physiologically relevant conditions (24). A recent publication also pointed toward a superiority of ADCC functions for anti-V2 bNAbs compared to other bNAbs when primary immune cells are used (63). These results differ from previously published data obtained using infected cell lines for quantifying ADCC (25), and support the relevance of a fully autologous ADCC system with infected primary target cells and NK effector cells (24, 63). Moreover, the data indicate that V2 epitopes may be particularly accessible on primary infected cells. Notably, CD4 expression was still detected on the primary T-cells infected with primary HIV isolates for 4 days demonstrating a limited down-regulation of CD4 expression compared to its almost complete disappearance observed on CEM.Nkr cell lines infected with the same viruses (66, 73). These differential CD4 expression patterns point to distinct CD4/trimeric Env engagement suggesting that epitopes such as the V2 loop may be more accessible to Abs on infected primary cells than on CEM.Nkr cell lines. The nature of the epitopes of the viral envelope glycoproteins exposed on the surface of infected primary cells requires further investigation.

Further comparison of infected primary cell lysis with other ADCC parameters shows that there is no strong correlation between lysis and binding of Abs to infected primary PBMCs or to CD107a down-regulation (24). Of note, the Abs tested in these and many other similar experiments are variably comprised of recombinant, hybridoma-derived monoclonal IgG, and polyclonal IgG isolated from vaccinated or infected patients. For the latter, Fc domains were therefore naturally induced, which is at variance with the Fc domains of most of the recent bNAbs where the V_H_, Vκ, and Vλ chains were sequenced and further reconstructed with defined heterogenous heavy chains, often using new proteomics approaches (74, 75). As the combination of the immunoglobulin heavy and light chains of the HIV-specific Abs may play a decisive role in ADCC, increased attention should be paid to the characterization of the Abs Fc domains, including post-translational modifications that may be specific to the native B cell, since they are essential for the induction of ADCC.

## Differing Value Propositions Offered by ADCC Assays

Collectively, these studies strongly underscore the need for additional comparative analyses (56) of all currently used ADCC assays, not only to better understand similarities and differences, but also to decipher the relevance of each assay relative to *in vivo* protection. For example, there is more to be learned by comparative testing in the context of vaccine and passive transfer studies in which efficacy has been observed. Indeed, there is a significant diversity of thought regarding the value of different approaches with respect to ability to support derivation of fundamental insights into host and virus interactions vs. the performance characteristics suitable for use in large-scale vaccine efficacy and immunogenicity studies. This divergence may largely reflect an inherent tradeoff between biological fidelity and practical scalability that poses a challenge to many fields. This spectrum of assays (Figure 2) and spectrum of differing utility is further intensified by conflicting observations among and differences in interpretations of data from clinical and NHP studies [reviewed in (76)]. However, continued investment in comparative and correlates studies promises the possibility of resolution.

## Biophysical Assays to Monitor Antibody Functionality in HIV Vaccine Trials

There are substantial challenges inherent to applying assessment of Fc-mediated effector function in cellular assays, particularly across large clinical studies. Cell-based assays of Fc-mediated functions, especially those that use frozen/thawed primary blood cells as targets or effectors, are relatively difficult to reproduce across diverse laboratories. Polymorphisms across effector cell FcRs can influence the outcome of cell-based assays; for example, the high affinity FcγRIIIa V158 allotypic variant is associated with more potent ADCC than the F158 variant with lower affinity for IgG (77, 78). Significant effort has therefore been directed toward developing biophysical assays that serve as useful proxies of Fc-mediated functions. Toward this end, several groups have developed and standardized methods to assess the FcR-binding capacity of antigen-specific Abs present in clinical samples (79–82). It is known that the affinities of the interaction between Ab and FcR are fundamental to Ab effector function, and as such, this parameter has long been a target of numerous successful molecular engineering efforts to increase or ablate effector functions (83, 84). FcR-mediated effector functions in general, and ADCC in particular, require the aggregation of FcR on the effector cell surface by IC. Leveraging the fact that multimeric FcR has a higher affinity for antigen-bound IgG than monomeric FcR, these biophysical approaches, namely FcγR dimer/multimer assays (79, 85), aim to mimic the capacity of a given antibody sample to form ICs that can avidly interact with FcR by assessing their capacity to interact with FcR multimers. The FcγR dimer/multimer assays typically exploit antigen-coated microwells, or multiplexed antigen-conjugated microbeads, which are then probed with immune sera, and bound Abs detected using multimeric FcRs (either dimers or tetramers) (79, 81). These assays have been shown to reliably reproduce the differences apparent among natural IgG types in binding to FcR relevant to Ab effector functions. For example, across a panel of monoclonal Ab variants, despite equivalent opsonization, the receptor binding profiles that drive the differing activities of the IgG subclasses and glycovariants were recapitulated *via* detection with multimerized FcR (79). Further, the FcγR dimer/multimer assays have been shown to be better correlated with effector function and more accurately predictive of the effector function of polyclonal responses than Ab titer in the context of influenza (81, 86, 87), and HIV (88–91). Further these assays are useful in modeling outcomes *in vivo* in the context of vaccination and natural infection (1, 60, 91–93). Common polymorphisms of FcRs can be studied in isolation and such analyses are consistent with the known function of such polymorphisms (e.g., the V/F158 polymorphism of FcγRIIIa and H/R131 polymorphism of FcγRIIa) (81, 85).

Biophysical assays of FcR engagement can be more sensitive and reproducible in comparison to cell-based assays of Fc-mediated functions. The simplicity and relatively low cost of biophysical assays mean these assays have become useful in probing the breadth of antigen recognition and breadth of FcRs bound, which may be important aspects of protective ADCC responses (85). In the setting of HIV vaccine response evaluations, recombinant proteins that properly capture antigen conformations relevant during infection (94) will make these assays more biologically relevant. As the field develops standardized panels of Env protein of diverse conformations, the biophysical assays of FcR engagement can be used to screen for breadth of Fc-functional Ab responses induced by vaccination. However, it is already known that biophysical binding assays can correlate well with multiple effector activities, for example, reflecting both the killing and trogocytosis components of the RFADCC assay (23, 85, 91). Lastly, biophysical assays are highly amenable to high-throughput analyses and correlations such as those employed for systems serology (95, 96). These advanced analytical tools offer a highly nuanced view of the differences or similarities between polyclonal responses present among different subjects/cohorts.

## Systematic Serology to Asses Other FcR-Mediated Effector Functions

Given this rich history of work developing ADCC assays and observations correlating these activities to outcomes in human and NHP studies, it is perhaps not surprising that effort to characterize this effector function has matured into similar efforts to assess other FcR-mediated effector functions (Figure 2). These activities include Ab-mediated phagocytosis carried out by monocytes, macrophages, and neutrophils (91, 97–101), antibody-dependent trogocytosis mediated by monocytes (23, 57, 102), as well as complement-dependent cytotoxicity (60, 82, 91, 103). Further, these activities extend all the way through to investigations of how Ab opsonization may impact antigen presentation, dendritic cell responses, and shape the development of germinal center reactions. Clearly, there is a wide spectrum of potential means by which Abs can mediate anti-viral activities, and yet, similar challenges confront assays of these activities, and because a number of these activities have also correlated with resistance to viral challenge (46, 60), similar questions as to the relevance of each *in vitro* assay to the processes that may contribute to *in vivo* outcomes exist.

In sum, the spectrum of FcγR-mediated effector function is extremely diverse as shown in recent systems serology studies that reveal the high dimensionality of interactions among FcγR classes, FcγR alleles, immunoglobulin classes, immunoglobulin subclasses, immunoglobulin glycosylation, and antigen specificity (96). By contrast, any single functional assay, such as for ADCC, samples only a subset of the many potential interactions. Thus, it is critical to reconcile observations made with this subset of interactions and a biological outcome, which underscores the importance of identifying ADCC assay formats that can be deployed in large scale HIV-1 vaccine trials that produce the essential biological data defining protection or its absence. Fortunately, the first rigorous comparative study of multiple methods to quantify different FcγR mediated effector functions, showed that four different ADCC assay formats produced data that was more highly concordant as compared with the other assays that were distinct from one another and ADCC (56). The clustering of ADCC data in that study strongly suggests the further development of assays that can be deployed in large-scale HIV-1 vaccine trials and natural history studies of Ab-mediated control of HIV-1 infection.

## The Complexity of Effector Cells for ADCC: Classical and Memory NK Cells

The classical NK cell subsets engaged by ADCC Ab responses were initially identified among Lineage negative, i.e., CD3-CD19-CD20-CD14-, human cells as those cells that express high level of CD16 receptor (CD16^high^) and simultaneously express low levels of CD56 (CD56^dim^) (104). More recently, other phenotypic characteristics of these cellular subsets have been identified such as co-expressing the NKG2D receptor (105) and being more differentiated to express CD57 (106). In the rhesus macaque, a commonly used NHP model for HIV-1 research, most of the NK cell subsets share analogous characteristics with their human counterparts for their ability to serve as ADCC effector cells (107).

In addition to the classic NK cell subsets, more recently NK cells with adaptive features have been described and could play a role as effector cells for ADCC responses. Memory-like NK cells are distinguished from other NK cell subsets by the following criteria: (1) they lack the gamma signaling chain of the FcγR and the Syk adaptor protein; (2) they still require Abs to grant antigen specificity; (3) they proliferate rapidly after antigen signaling; and (4) they are more potent mediators of ADCC (35, 36). These cells, designated as FcγRΔg NK cells, are massively expanded by CMV infection. Recent data now shows that FcγRΔg NK cells are also present in rhesus macaques where they are also expanded by rhesus CMV positivity (108). Further, FcγRΔg NK cells are distributed in peripheral tissues, particularly enriched in the mucosae, and their frequencies are increased in lymphoid tissues in SIV-infected animals. The nature of FcγRIIIa signaling in FcγRΔg NK cells is clearly distinct from other NK cell subsets and is mediated through the CD3ζ chain, accounting, at least partially, for the enhanced functions. Collectively, the available data suggest that FcγRΔg NK cells are strong candidates as effector cells for ADCC *in vivo* setting the stage to determine how they impact Ab-mediated protection against HIV-1.

## Caveats

Although a wealth of data spanning mouse and NHP models to human studies suggests the relevance of Ab effector functions, including ADCC, to anti-viral activity *in vivo*, it is important to note that many of these studies are often by nature associational and cannot clearly delineate mechanistic relevance. Similarly, NHP studies often rely on small cohorts resulting in limitations in the ability to confidently assess relationships (or lack thereof) between assays and outcomes; there are studies in which ADCC activity but not protective efficacy was observed (109, 110), as well as vaccines and passive antibody transfer experiments that have shown protection not associated with ADCC (111, 112). In rhesus macaques, passive monoclonal Ab transfer experiments have suggested the importance of effector function, but have not allowed conclusive determination of whether non-neutralizing Abs might be sufficient to provide protection, or indicated that enhancing the ADCC activity of a monoclonal Ab can result in improved protection (111, 113–115) as strongly as similar studies conducted in mouse models have (116–118). Further, the ways in which effector cells (119), Ab receptors (120), and Ab types (121, 122) present in model systems differ from those in humans introduce a number of potentially confounding factors. Differences in viruses and mode of challenge further compound challenges in translation. Even among human studies, it is worth noting that ADCC was identified in secondary analysis of a vaccine with a low level of efficacy, and mother to child transmission studies are few in number and need to be repeated in additional cohorts. Thus, it is worth remembering that while use of various assays allows for exciting exploration of relevant aspects of Ab and effector immunology and HIV virology at great resolution and with many nuances, considerable *in vivo* knowledge gaps remain.

## Conclusions

The complex mechanism of ADCC makes its *in vitro* detection highly challenging. Its mechanistic relationships with *in vivo* protection are yet to be defined. Nonetheless, numerous assays have been developed to dissect this phenomenon. The data obtained by these assays has contributed to our ever-increasing knowledge on the role of ADCC in HIV/AIDS. Future studies need to investigate other potential ADCC parameters including the HIV epitopes accessible on the target cells; the role of Ab isotype, specific Fc domains, as well as the FcR counterpart expression and function on the effector cells in relevant tissues; and the potential of various effector cells to induce target cell lysis. An increased knowledge of parameters implicated in ADCC functions is a prerequisite for a better understanding of its potential role *in vivo*. Such information will allow us to gain insight and knowledge for future HIV vaccine development.

## Author Contributions

All authors listed have made a substantial, direct and intellectual contribution to the work, and approved it for publication.

### Conflict of Interest Statement

The authors declare that the research was conducted in the absence of any commercial or financial relationships that could be construed as a potential conflict of interest.
